# Relationship Between Pulmonary Artery Pressure and Inflammation Parameters

**DOI:** 10.7759/cureus.52427

**Published:** 2024-01-17

**Authors:** Sabri Abuş

**Affiliations:** 1 Cardiology, Adıyaman University, Adıyaman, TUR

**Keywords:** c-reactive protein to albumin ratio, inflammation, pulmonary artery pressure, congestive heart failure, pulmonary hypertension

## Abstract

Background

Inflammation can play a role in the development and progression of pulmonary hypertension (PHT). In this study, inflammatory parameters were compared in congestive heart failure (CHF) patients with and without PHT. The relationship between pulmonary artery pressure (PAP) and inflammatory parameters was investigated.

Materials and methods

Out of 80 CHF patients, 40 had PHT. The patients' age, gender, smoking status, comorbidities such as diabetes mellitus (DM) and hypertension (HT), and mortality rates were recorded. Inflammatory parameters were recorded.

Results

The mean age of the PHT group was 64.38 ± 9.17 and the mean age of the non-PHT group was 64.70 ± 8.99. There were 23 men and 17 women in the PHT group, and there were 21 men and 19 women in the non-PHT group. There was no significant difference between the two groups in terms of mean age and gender distribution (p = 0.874 and p = 0.653). Accordingly, the C-reactive protein to albumin ratio (CAR) value was statistically significantly higher in PHT patients (p = 0.023). The eosinophil count was found to be significantly higher in non-PHT patients (p = 0.015). Accordingly, a significant correlation was detected between CAR and PAP (r = 0.392 and p < 0.001).

Conclusion

In this study, the positive correlation between PAP and CAR and the significantly higher CAR value in PHT patients indicate the presence of inflammation in PHT patients. Studies on the relationship between inflammation and mortality in PHT patients may contribute more to the literature in the future.

## Introduction

Pulmonary hypertension (PHT) refers to elevated blood pressure in the pulmonary arteries, which are the vessels that carry blood from the heart to the lungs for oxygenation. PHT can be idiopathic or secondary to other conditions such as heart or lung diseases, blood clots in the lungs, or chronic obstructive pulmonary disease [1]. Shortness of breath, fatigue, chest pain, dizziness, and fainting are common symptoms of PHT. If left untreated, PHT can lead to right-sided heart failure, where the right side of the heart struggles to pump blood to the lungs, causing further complications [2].

Congestive heart failure (CHF) occurs when the heart is unable to pump blood efficiently, leading to a buildup of fluid in the lungs and other tissues. Common causes include coronary artery disease, heart attacks, hypertension, valvular heart disease, and cardiomyopathy. Symptoms of CHF include shortness of breath, fatigue, swelling in the legs and abdomen, and difficulty exercising. Over time, CHF can lead to damage and dysfunction in the heart muscle, further worsening the pumping ability of the heart [3].

PHT can cause increased pressure in the pulmonary arteries, leading to strain on the right side of the heart. Over time, this can contribute to right heart failure. In some cases, PHT secondary to lung diseases can lead to a condition known as cor pulmonale, where the right ventricle of the heart becomes enlarged and weakened [4]. Managing patients with both PHT and CHF can be challenging as medications that dilate pulmonary arteries may worsen fluid retention. Careful balance is required in the selection of medications [5].

Inflammation can play a role in the development and progression of PHT. While the exact mechanisms are complex and not fully understood, inflammation is believed to contribute to the remodeling of the pulmonary arteries, leading to increased pressure in the pulmonary circulation [6]. A number of events contribute to the pathogenesis of PHT, beginning with the degeneration of pulmonary artery endothelial cells brought on by different stressors. This is followed by pulmonary artery spasm, adhesion and migration of inflammatory cells to the pulmonary artery wall, adventitial fibrosis, intimal occlusive fibrosis, and fibrinoid necrosis, finally leading to the development of plexiform lesions. PHT is the consequence of an intricate and intriguing interaction of several inflammatory factors [7].

In this study, inflammatory parameters were compared in CHF patients with and without PHT. The relationship between pulmonary artery pressure (PAP) and inflammatory parameters was investigated.

## Materials and methods

Study design

The University of Adıyaman's Academic Ethical Review Committee approved the research technique (Approval date: 2023-12-19; IRB Number: 2023/4-14). Every participant completed a permission form for the research to be conducted. The study method was conducted in accordance with the Declaration of Helsinki. The study is descriptive and cross-sectional in character.

Study group

Out of 80 CHF patients, 40 had PHT. The patients' age, gender, smoking status, comorbidities such as diabetes mellitus (DM) and hypertension (HT), and mortality rates were recorded.

Echocardiographic examination

Transthoracic echocardiographic examinations were performed in all subjects using Vivid 5S (GE Healthcare Systems, Horten, Norway) with a 2.0-3.5 MHz transducer. PAP and ejection fraction (EF) were recorded on echocardiography.

Laboratory examination

A venous blood sample was obtained upon hospital admission. To quantify white blood cells (WBC), including neutrophils and lymphocytes, Abbott Diagnostics (Abbott Park, IL, USA) employed the CELL-DYN Ruby computerized hematology testing instrument. Additionally, the counts of hemoglobin, albumin, and platelets were ascertained. To calculate the neutrophil-to-lymphocyte ratio (NLR), the number of neutrophils is divided by the number of lymphocytes. To calculate the monocyte-to-lymphocyte ratio (MLR), the number of monocytes is divided by the number of lymphocytes. To calculate the platelet-to-lymphocyte ratio (PLR), the number of platelets is divided by the number of lymphocytes. The C-reactive protein (CRP) to albumin ratio (CAR) was measured. The following is the calculation of the pan immunological inflammatory value (PIV), which was based on precise measurements of total blood concentrations:

PIV = [neutrophils (10^6^/mm^3^) × platelets (10^3^/mm^3^) × monocytes (10^3^/ mm^3^)]/lymphocytes (10^3^/mm^3^).

To compute the systemic immune inflammation index (SII), the following equation was used:

SII = [neutrophils (10^6^/mm^3^) × platelets (10^3^/mm^3^)]/lymphocytes (10^3^/mm^3^).

Statistical examination

Using SPSS version 26.0 software (IBM Corp., Armonk, NY), data evaluation was done. A Kolmogorov-Smirnov test analysis was conducted to identify patterns in the data. Mann-Whitney U tests were used for discontinuous numerical variables, and independent sample t-tests were used for numerical variable comparisons. To compare qualitative factors within the research group, chi-square tests were used. Continuous variables complying with normal distribution were shown as mean ± standard deviation. Continuous variables that did not comply with normal distribution were shown as median (minimum-maximum). Categorical variables were expressed as numbers and percentages. Spearman correlation test was used to examine the relationship between variables. P < 0.05 was accepted as a statistical significance value.

## Results

The sociodemographic status, echocardiography results, and comorbidities of the PHT and non-PHT groups are shown in Table 1. The mean age of the PHT group was 64.38 ± 9.17 and the mean age of the non-PHT group was 64.70 ± 8.99. There were 23 men and 17 women in the PHT group, and there were 21 men and 19 women in the non-PHT group. There was no significant difference between the two groups in terms of mean age and gender distribution (p = 0.874 and p =0.653). Although mortality was found to be higher in PHT patients, the difference was not statistically significant (p = 0.056). Although DM and HT were more common in PHT patients, the difference was not significant (p = 0.655 and p = 0.251). EFs did not vary between groups (p = 0.822). PAP value was 41.38 ± 10.99 in the PHT group and 19.70 ± 3.40 in the non-PHT group.

**Table 1:** Comparison of sociodemographic and echocardiography parameters of patients diagnosed with CHF. * P < 0.05 was accepted as a statistical significance value. Mann-Whitney U test and chi-square test were used. The data were represented as n (%) or median (minimum-maximum). CHF: congestive heart failure; PHT: pulmonary hypertension; DM: diabetes mellitus; HT: hypertension; EF: ejection fraction; PAP: pulmonary artery pressure.

Variable		PHT (n = 40), (n, %, or median (min.-max.)	Non-PHT (n = 40), (n, %, or median (min.-max.)	p
Age (years)		66 (44-77)	64 (45-76)	0.874
Gender (n/%)	Female	17	19	0.653
	Male	23	21	
Mortality (n/%)		12 (30)	5 (12.5)	0.056
Smokers (n/%)		16 (40)	19 (47.5)	0.499
DM (n/%)		21 (52.5)	19 (47.5)	0.655
HT (n/%)		27 (67.5)	22 (55)	0.251
EF, %		33 (25-38)	34 (24-39)	0.822
PAB, mmHg		40 (26-65)	20 (13-24)	<0.001*

The hemogram and inflammation parameters of the groups are shown in Table 2. Accordingly, the CAR value was statistically significantly higher in PHT patients (p = 0.023). The eosinophil count was found to be significantly higher in non-PHT patients (p = 0.015).

**Table 2:** Comparison of inflammatory parameters of patients diagnosed with CHF. * P < 0.05 was accepted as a statistical significance value. Independent t-test and Mann-Whitney U test were used. The data were represented as mean ± SD or median (minimum-maximum). CHF: congestive heart failure; PHT: pulmonary hypertension; CRP: C-reactive protein; WBC: white blood cells; NLR: neutrophil-to-lymphocyte ratio; MLR: monocyte-to-lymphocyte ratio; PLR: platelet-to-lymphocyte ratio; CAR: CRP to albumin ratio; SII: systemic immune inflammation index; PIV: pan immune inflammation value.

Variables	PHT (n = 40), mean ± SD or median (min.-max.)	Non-PHT (n = 40), mean ± SD or median (min.-max.)	p
Hemoglobin, mg/dL	15.35 (9.62-17.80)	14.75 (10.05-16.68)	0.637
Albumin, mg/dL	4.30 (3.90-5.30)	4.40 (3.80-5.00)	0.138
CRP, mg/dL	0.20 (0.10-0.30)	0.20 (0.10-0.22)	0.177
WBC, 10^3^/μL	6.99 (4.46-11.70)	7.85 (5.03-12.30)	0.099
Neutrophil, 10^6^/μL	4.20 (2.06-8.16)	4.01 (1.96-7.89)	0.597
Lymphocyte, 10^3^/μL	2.27 ± 0.82	2.54 ± 0.71	0.123
Monocyte, 10^3^/μL	0.48 (0.13-1.10)	0.55 (0.28-1.10)	0.220
Eosinophil, 10^3^/μL	0.11 (0.01-0.64)	0.21 (0.02-0.71)	0.015*
Basophil, 10^3^/μL	0.037 (0.00-0.15)	0.04 (0.00-0.40)	0.567
Platelet, 10^3^/μL	249.46 ± 69.57	242.83 ± 75.84	0.685
NLR	1.87 (0.77-5.60)	1.78 (0.71-5.59)	0.679
MLR	0.24 ± 0.11	0.24 ± 0.09	0.829
PLR	122.18 (50.20-223.94)	88.78 (42.61-259.88)	0.098
CAR	0.048 (0.02-0.07)	0.046 (0.02-0.05)	0.023*
SII	467.53 (178.45-1553.75)	436.02 (118.70-1392.96)	0.436
PIV	193.72 (62.64-1333.12)	215.86 (39.17-1026.22)	0.765

The possible correlation of inflammatory parameters and EF with PAP is shown in Table 3. Accordingly, a significant correlation was detected between CAR and PAP (r = 0.392 and p < 0.001).

**Table 3:** Inflammatory parameters and the relationship between EF and PAP. * P < 0.05 was accepted as a statistical significance value. Spearman correlation test was used. PAP: pulmonary artery pressure; EF: ejection fraction; NLR: neutrophil-to-lymphocyte ratio; MLR: monocyte-to-lymphocyte ratio; PLR: platelet-to-lymphocyte ratio; CAR: C-reactive protein to albumin ratio; SII: systemic immune inflammation index; PIV: pan immune inflammation value.

Variables	PAP
EF	r = 0.182
	p = 0.106
CAR	r = 0.392
	p < 0.001*
SII	r = 0.112
	p = 0.322
PIV	r = 0.024
	p = 0.835
NLR	r = 0.108
	p = 0.339
MLR	r = 0.010
	p = 0.931
PLR	r = 0.103
	p = 0.362

## Discussion

Inflammatory mediators, such as cytokines and growth factors, may be released in response to various stimuli, contributing to inflammation in the pulmonary arteries. These mediators can promote processes like vasoconstriction, cell proliferation, and fibrosis, which can lead to the thickening and narrowing of the pulmonary vessels. Immune system dysregulation and inflammatory responses may be involved in the development of certain types of PHT, especially in conditions like connective tissue diseases and autoimmune disorders. Inflammatory cells, such as macrophages and T lymphocytes, may infiltrate the pulmonary vascular wall, contributing to vascular remodeling [8].

Inflammation can lead to dysfunction of the endothelial cells lining the pulmonary arteries. Endothelial dysfunction is a key factor in the development of PHT. Dysfunctional endothelial cells may release substances that promote vasoconstriction and cell proliferation, contributing to increased pulmonary vascular resistance. Underlying chronic inflammatory conditions, such as rheumatoid arthritis or inflammatory lung diseases, can be associated with an increased risk of developing PHT [9].

Inflammation can contribute to the formation of blood clots in the pulmonary arteries, leading to pulmonary embolism. Chronic thromboembolic pulmonary hypertension is a type of PHT that can result from unresolved pulmonary emboli. Some medications used to treat PHT, such as prostacyclin analogs and endothelin receptor antagonists, may have anti-inflammatory effects and can help mitigate vascular remodeling [10].

It is important to note that the relationship between inflammation and PHT is multifaceted, and research in this area is ongoing. Understanding the inflammatory processes involved in PHT may lead to the development of targeted therapies to address these specific mechanisms and improve treatment outcomes. Individuals with suspected or diagnosed PHT should work closely with their healthcare providers for a comprehensive evaluation and appropriate management [11].

CAR is a laboratory marker that has been studied in various medical conditions, including inflammatory and cardiovascular diseases. CRP is an acute-phase protein produced by the liver in response to inflammation, while albumin is a protein that serves multiple functions, including maintaining oncotic pressure in the blood [12]. In a study conducted on acute coronary syndrome, serum albumin value was found to be significantly low in patients with total occlusion of the coronary arteries. In the same study, CRP was found to be higher in patients suffering from acute coronary syndrome [13]. In a study conducted in patients with three-vessel disease, it was observed that a high fibrinogen-albumin ratio predicted mortality [14]. Albumin value was found to be significant in patients who died from COVID-19 [15].

CAR is often used as an inflammatory marker, and an elevated ratio may indicate increased systemic inflammation. Elevated levels of CRP have been associated with various cardiovascular conditions, including PHT. However, it is essential to interpret this ratio in the context of the overall clinical picture, as multiple factors can influence CRP levels [16]. Elevated CAR has been associated with worse outcomes in various cardiovascular and inflammatory conditions [17-19]. However, the specific use of this ratio in the context of PHT may require further research for validation and standardization.

PHT can have a significant impact on health and may lead to increased mortality, especially if not properly managed. The prognosis and mortality rates associated with PHT can vary based on several factors, including the underlying cause, severity of the condition, and the presence of other coexisting health issues [20].

In their study conducted in 2022, Çerik et al. compared 72 PHT patients with 99 non-PHT controls. Accordingly, they found CAR, NLR, and PLR values to be significantly higher in PHT patients. When they compared the PHT patients who died during follow-up and the PHT patients who survived, they found the NLR, MLR, and CAR values of the PHT patients who died to be significantly higher. They also found that CAR predicted mortality in PHT patients [21].

Quarck et al. reported that high CRP levels in PHT patients decreased after treatment. It also found that high CRP levels were associated with mortality [22]. Özpelit et al. reported that NLR predicts mortality in PHT patients, and as the NLR level increases, the CRP value also increases [23].

This study has some limitations. The limited number of participants, single-center nature, and cross-sectional nature of the study are important limitations.

## Conclusions

Although there are studies on inflammation in PHT in the literature, there are no studies on the relationship between PHT and mortality rate in CHF patients and the relationship between inflammatory parameters and PAP. In this study, the positive correlation between PAP and CAR and the significantly higher CAR value in PHT patients indicate the presence of inflammation in PHT patients. Studies on the relationship between inflammation and mortality in PHT patients may contribute more to the literature in the future.
